# XPO1 inhibitors represent a novel therapeutic option in Adult T-cell Leukemia, triggering p53-mediated caspase-dependent apoptosis

**DOI:** 10.1038/s41408-021-00409-3

**Published:** 2021-02-01

**Authors:** Eline Boons, Tatiane C. Nogueira, Tim Dierckx, Soraya Maria Menezes, Maarten Jacquemyn, Sharon Tamir, Yosef Landesman, Lourdes Farré, Achiléa Bittencourt, Keisuke Kataoka, Seishi Ogawa, Robert Snoeck, Graciela Andrei, Johan Van Weyenbergh, Dirk Daelemans

**Affiliations:** 1 KU Leuven Department of Microbiology, Immunology and Transplantation, Laboratory of Virology and Chemotherapy, Rega Institute, B-3000 Leuven, Belgium; 2 KU Leuven Department of Microbiology, Immunology and Transplantation, Laboratory of Clinical and Epidemiological Virology, Rega Institute, B-3000 Leuven, Belgium; 3 Karyopharm Therapeutics, Newton, MA USA; 4 Instituto de Pesquisa Goncalo Moniz, Oswaldo Cruz Foundation (FIOCRUZ), Salvador, Bahia Brazil; 5 HUPES, Federal University of Bahia (UFBA), Salvador-Bahia, Brazil; 6 Department of Pathology and Tumor Biology, Kyoto University, Kyoto, Japan

**Keywords:** Leukaemia, Tumour virus infections, Targeted therapies

Dear Editor,

Human T-cell Leukemia Virus type 1 (HTLV-1) is the etiological agent of adult T-cell leukemia (ATL). Although the majority of HTLV-1-infected individuals remain asymptomatic, in endemic regions such as Southern Japan, the Caribbean, some parts of Oceania, Romania, Central and South America, Northern Iran, and Central Africa, up to 4% of people living with HTLV-1 develop ATL. Although combination therapy with IFN-α and AZT as a first-line treatment prolongs survival of patients with ATL significantly, prognosis for patients with aggressive forms of ATL is poor due to intrinsic chemoresistance and relapse^1^.

The viral proteins Tax and HBZ play a major role in HTLV-1-induced carcinogenesis^2^. Tax drives the ATL epigenetic signature through the NF-κB pathway and inactivates the tumor-suppressor protein p53^3–5^. Inhibition of exportin-1 (CRM1/XPO1), the key nuclear export factor for proteins containing the typical leucine-rich nuclear export signal (NES), has been shown to inhibit NF-κB activity and induce p53-signaling pathways^6^, whereas XPO1 inhibitors display efficacy against different types of cancer^7–9^. Recently, the first-in-class XPO1 inhibitor selinexor (XPOVIO^®^) has been approved by the US Food and Drug Administration for the treatment of relapsed and refractory multiple myeloma and for relapsed diffuse large B-cell lymphoma. Selinexor is a highly selective and covalent inhibitor of XPO1 preventing export of cargo proteins to the cytoplasm, resulting in nuclear accumulation of cargo proteins^10–12^. Here, we investigate the therapeutic potential of selinexor in ATL by combining a two-stage targeted and systems analysis of ex vivo ATL transcriptomes with functional validation HTLV-1-transformed CD4+ cells.

Since ATL leukemic cells are characterized as CD4^+^CD25^+^CADM1^+^, we first investigated the possible relationship of XPO1 to these three signature genes, as well to p53 and NFκB signaling, which are the major regulators of apoptosis and survival in ATL^13–15^. As shown in Fig. 1A, ATL patients display an XPO1^hi^ phenotype as compared to healthy controls, which was significantly and positively correlated to ATL leukemic markers CD4, CD25/IL2RA and CADM1/TSCL1 in two independent patient cohorts from different HTLV-1 endemic areas (Brazilian cohort^13–15^, *n* = 9; Fig. 1C and Supplementary Fig. 1 for Japanese cohort^13–15^, *n* = 44). In contrast, a strong negative correlation between *XPO1* and *IL2RA* was observed in PBMCs from healthy controls in the Brazilian cohort (Fig. 1A), which we validated in purified CD4+ cells from a large independent cohort of healthy controls (Supplementary Fig. 1, r = −0.23, *p* < 0.0001, *n* = 294). This suggests a pathobiological role for *XPO1* in ATL leukemogenesis. Likewise, increased *XPO1* transcript levels were found to be associated with clinical progression to aggressive ATL (Fig. 1B). Noteworthy, *XPO1* expression neither differed between Hbz-high expressing (>10 transcripts per million, TPM) or Hbz-low expressing (<10 TPM) ATL patients, nor was there any difference in *XPO1* expression between Tax-positive and Tax-negative ATL patient samples (*p* > 0.7 for both, Fig. 1B, inset). Furthermore, the combined XPO1^hi^IL2RA^hi^ phenotype was unique to ATL transcriptomes, as it was absent in other acute lymphocytic leukemias (B-ALL and T-ALL, Supplementary Fig. 1). Therefore, we proceeded to a genome-wide systems analysis of XPO1 in ATL, using a modular approach to integrate molecular, cellular and clinical data from both ATL cohorts. Whole Genome Correlation Network Analysis (WGCNA) of transcriptome data identified 31 unique gene modules in two independent ATL cohorts and confirmed *XPO1* belongs to the same transcriptional module as leukemic marker *CADM1* (Fig. 1C). Moreover, WGCNA also confirmed *CD4* as a significant member of the *XPO1* gene module in both cohorts (*p* = 0.018 and *p* = 1.45 ×10^−5^, data not shown). In agreement with this finding, we observed a significant positive correlation between *XPO1* and *CD4* transcript levels (a proxy for total CD4^+^ leukemic cells) and a negative correlation for *CD8B* transcripts (a proxy for antileukemic CD8^+^ cells) in the Japanese cohort, which we confirmed by flow cytometry in the Brazilian cohort (Fig. 1C). Hinting at a possible link to apoptosis, the XPO1 gene module comprised *TP53* (the gene encoding apoptosis regulator p53) and several proapoptotic caspases (“executors” *CASP3* and *CASP7*, “initiator” *CASP10*, all *p* < 0.05). As a negative control, we found that inflammatory caspases *CASP1, CASP4*, and *CASP5* were not correlated to the *XPO1* gene module (*p* > 0.30 in both cohorts, not shown). *XPO1* transcript levels were also not correlated to gender (not shown), age, or patient survival (Fig. 1C), arguing against a possible selection bias in ATL patients that might drive the observed XPO1 results. However, we found a a significant positive correlation of *CASP3* (r = 0.741, *p* = 0.03) and *CASP7* (r = 0.84, *p* < 0.01) with age at ATL diagnosis, suggesting that apoptosis might be protective in vivo by leading to later disease onset. In addition, “executor” caspase *CASP7* (r = 0.82, *p* = 0.01) was positively correlated to ex vivo apoptosis, measured by short-term culture of unstimulated patient PBMCs. In agreement with a protective role for apoptosis in ATL, *CASP10* levels showed a trend of correlation to longer survival (r = 0.64, *p* = 0.08), which remains to be investigated in larger cohorts. Unfortunately, no functional assay or survival data were available for the larger Japanese cohort.

**Fig. 1:**
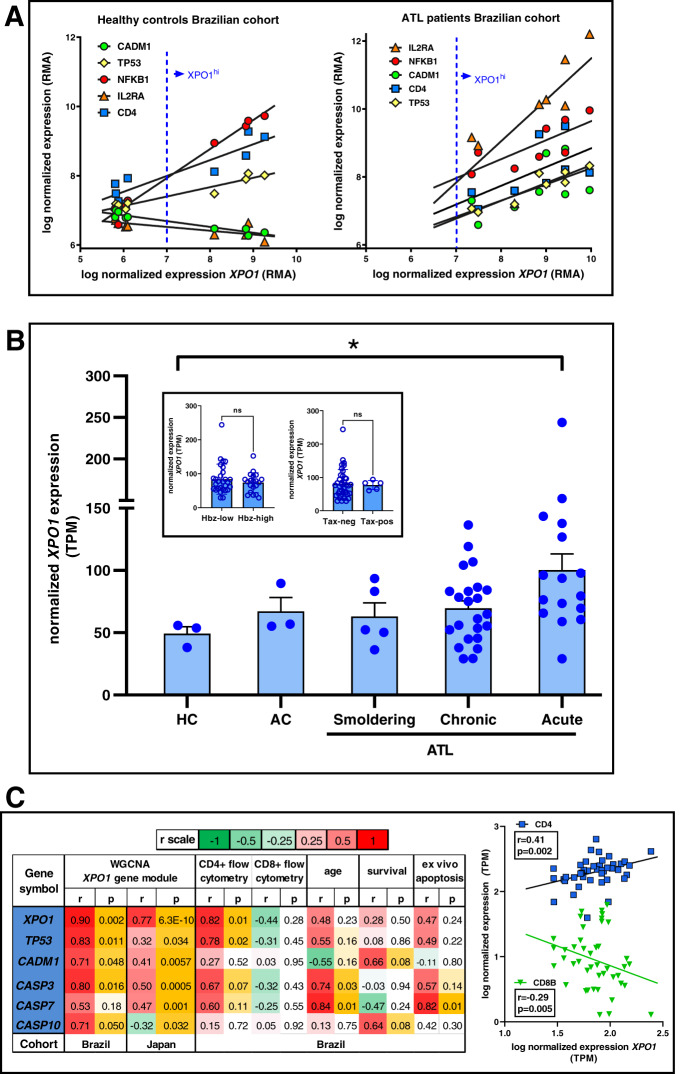
*XPO1* transcripts positively correlate to leukemic markers *CD4/IL2RA/CADM1, TP53/NKFB1* transcripts and clinical progression in Adult T-cell Leukemia patients. **A** *XPO1* transcripts were analyzed by transcriptome-wide correlation in PBMC from healthy controls and Adult T-cell leukemia patients from the Brazilian cohort. Data are expressed as RMA (Robust Multi-array Average). Spearman correlation was used (all *p* < 0.05). **B** *XPO1* levels increase with clinical progression to aggressive ATL. *XPO1* transcript levels were quantified in matched samples from the Japanese cohort: healthy controls (HC), asymptomatic carriers (AC), and patients with smoldering, chronic and acute ATL (*p < 0.05 ANOVA with post-test for linear trend). Inset: *XPO1* expression does not differ between Hbz-high expressing (>10 transcripts per million, TPM) or Hbz-low expressing (<10 TPM) ATL patients, or between Tax-positive and Tax-negative ATL patient samples (*p* > 0.7 for both). **C** Whole Genome Correlation Network Analysis (WGCNA) demonstrates *XPO1* belongs to the same transcriptional module as leukemic marker *CADM1*, apoptosis regulators *TP53* and caspases (*CASP3-7-10*). Spearman test was used for correlation with WGCNA gene module ‘eigengene’ (confirming the similarity in the *XPO1* gene module in both Brazilian and Japanese cohorts) and patient data (age, survival, flow cytometry, ex vivo apoptosis measured after short-term culture of primary cells) for the Brazilian cohort (left panel) and *CD4* and *CD8B* transcripts for the Japanese cohort (right panel).

*XPO1* expression is strongly correlated to *NFKB1* and *TP53* transcript levels (Fig. 1A–C), which are master regulators of cell survival and cell death in ATL, respectively, as has been extensively demonstrated both in vitro and in vivo^13–15^. Since obtaining fresh ATL leukemic cells of sufficient quality and quantity is a limiting factor for mechanistic in vitro studies, we used HTLV-1-transformed CD4 + T-cell lines (MT-2 and MT-4) to elucidate the effect of XPO1 inhibition by selinexor (KPT-330, XPOVIO^®^) upon p53 signaling and downstream apoptosis. As shown in Fig. 2A, selinexor strongly induced p53 upregulation as well as phosphorylation, in a dose-dependent manner, in both MT-2 and MT-4 cell lines. Selinexor treatment also caused nuclear accumulation of both p53 and IκB, as both proteins are indeed known cargo of XPO1 (Supplementary Fig. 2A). Nuclear p53 accumulation (Supplementary Fig. 2B) was accompanied with a functional p53 response as evidenced by upregulation of its downstream effector Bax (Supplementary Fig. 2C). In parallel with p53 activation, selinexor treatment of MT-2 and MT-4 cell lines resulted in increased cell death (Fig. 2B), mediated by apoptosis, as suggested by annexinV/PI staining (Fig. 2C). We confirmed the apoptotic nature of selinexor-induced cell death by western blot analysis of PARP cleavage and caspase-3 activation (Supplementary Fig. 2D). Following up on the ex vivo results obtained for *CASP3, CASP7*, and *CASP10* in ATL patients, we found that a pan-caspase inhibitor Q-VD-OPh (targeting caspases 3-7-8-9-10-12) was able to completely block PARP cleavage and caspase-3 activation (Supplementary Fig. 2D), as well as apoptotic cell death (not shown). Selinexor treatment also caused a significant decrease in XPO1 protein levels (Supplementary Fig. 2E).

**Fig. 2:**
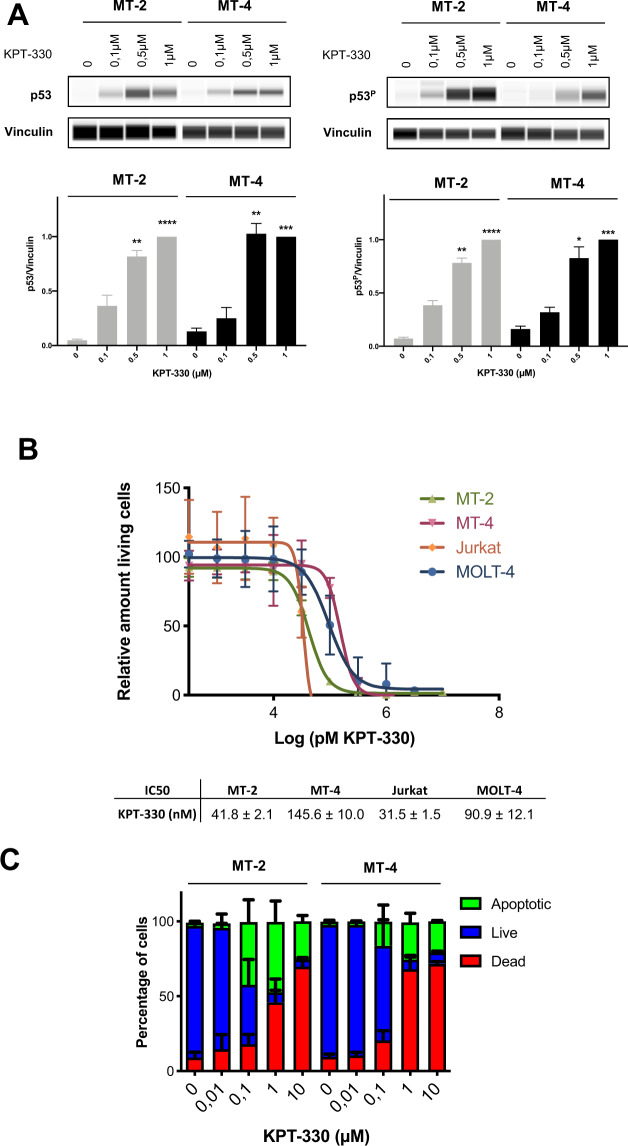
Pharmacological inhibition of XPO1 induces cell death, p53 upregulation/phosphorylation, and caspase-dependent apoptosis, in HTLV-1-transformed cell lines. **A** Simple western analysis for p53 and phosphorylated p53 was performed on whole-cell lysates after overnight incubation of MT-2 or MT-4 cells with different concentrations of KPT-330. The graphs show mean of p53 and phosphorylated p53 expression relative to vinculin expression with SEM (*n* = 4). Data are normalized to 1 µM KPT-330 within each separate experiment. RM one-way ANOVA, with Geisser-Greenhouse correction. *p = 0.0332; **p = 0.0021, ***p = 0.0002, and ****p < 0.0001. **B** Cell viability relative to untreated control as determined by MTT of HTLV-1 transformed cell lines in the presence of different concentrations KPT-330. Error bars represent standard error of mean (*n* = 3). **C** MT-2 or MT-4 cells were treated with DMSO or different concentrations of KPT-330 and after 3 days analyzed by Annexin V/PI flow cytometry. Error bars represent standard error of mean (SEM) (*n* = 3).

We and others have previously demonstrated that a combined in vitro, ex vivo and in silico approach might recapitulate the ATL in vivo response^16–19^. In addition, we have also shown that HTLV-1-transformed MT-2 and MT-4 cells phenocopy primary ATL cells^18^ in their relative resistance to the antiproliferative and proapoptotic effects of IFN-α^16,17^. Analogous to our findings with selinexor, we previously demonstrated that the significant antiproliferative and proapoptotic effect of IFN-β, but not IFN-α, was linked to increased p53 signaling in primary ATL cells^16^.

Of note, the *XPO1* gene module we identified in ATL patients also contained *IRF4* (data not shown), which has been identified as a sensitive predictor of IFN + AZT therapy failure^20^. Therefore, IRF4-overexpressing patients^21^ likely to fail first-line therapy might preferentially benefit from XPO1 targeting.

In conclusion, this study demonstrates that pharmacological inhibition of XPO1 by selinexor is a potential novel therapeutic strategy in ATL, by triggering the proapoptotic p53 pathway leading to caspase-dependent apoptosis. With selinexor (XPOVIO^®^) currently already available for the treatment of multiple myeloma and DLBCL and under investigation in multiple clinical trials for hematological and solid malignancies, these data provide a strong rationale for further therapeutic evaluation of selinexor to improve the outcome of patients with ATL.

## Supplementary information

Supplemental Figures
