# Supplementary material for: XPO1 inhibitors represent a novel therapeutic option in Adult T-cell Leukemia, triggering p53-mediated caspase-dependent apoptosis
Source: Blood Cancer J. 2021 Feb 1;11(2):27. doi: 10.1038/s41408-021-00409-3 (PMC7873181; doi:10.1038/s41408-021-00409-3)
Supplement: Supplementary file 1 — Supplemental Figures [file 41408_2021_409_MOESM1_ESM.pptx]

## Slide 1
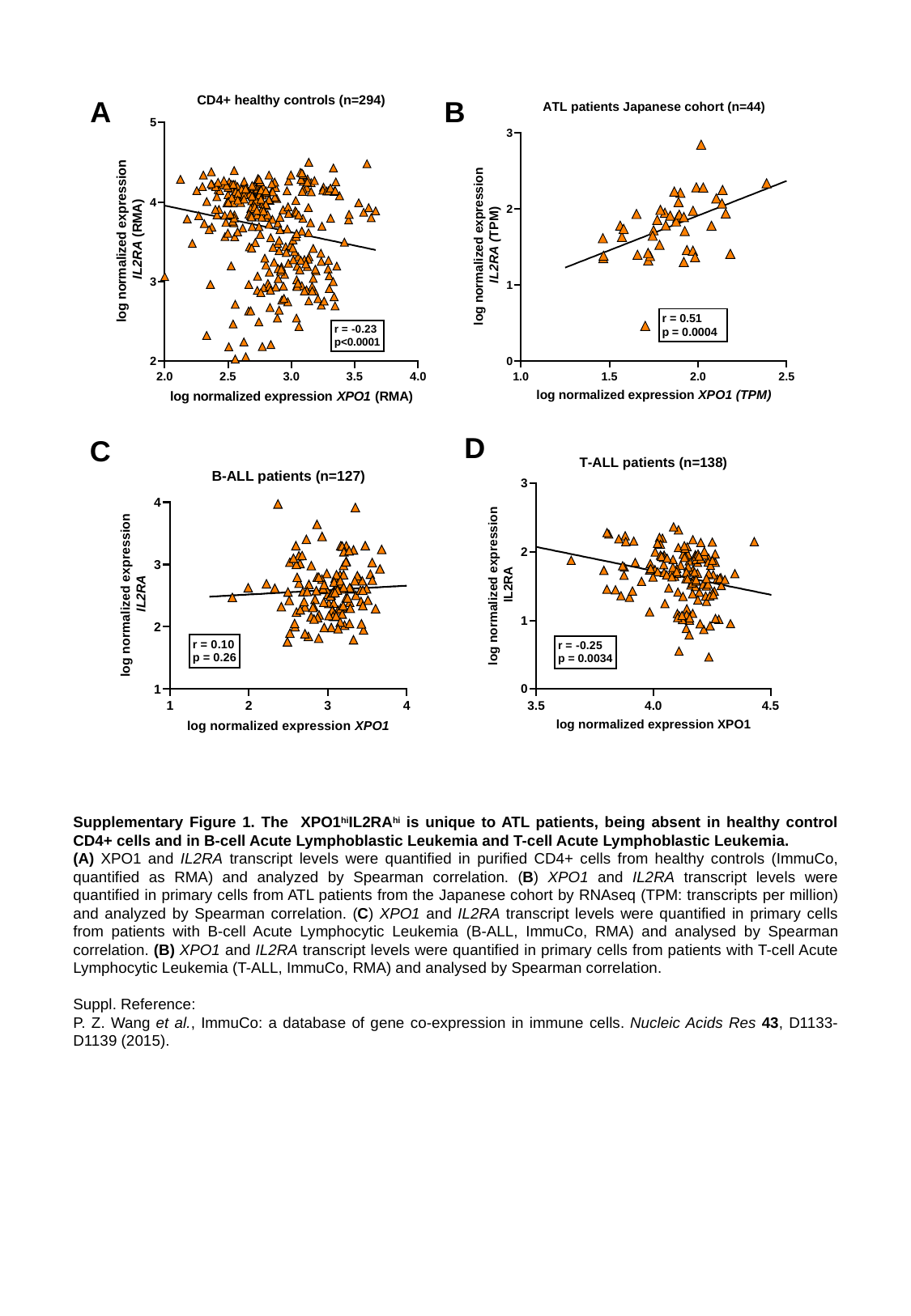

Supplementary Figure 1. The XPO1hiIL2RAhi is unique to ATL patients, being absent in healthy control CD4+ cells and in B-cell Acute Lymphoblastic Leukemia and T-cell Acute Lymphoblastic Leukemia.
(A) XPO1 and IL2RA transcript levels were quantified in purified CD4+ cells from healthy controls (ImmuCo, quantified as RMA) and analyzed by Spearman correlation. (B) XPO1 and IL2RA transcript levels were quantified in primary cells from ATL patients from the Japanese cohort by RNAseq (TPM: transcripts per million) and analyzed by Spearman correlation. (C) XPO1 and IL2RA transcript levels were quantified in primary cells from patients with B-cell Acute Lymphocytic Leukemia (B-ALL, ImmuCo, RMA) and analysed by Spearman correlation. (B) XPO1 and IL2RA transcript levels were quantified in primary cells from patients with T-cell Acute Lymphocytic Leukemia (T-ALL, ImmuCo, RMA) and analysed by Spearman correlation.
Suppl. Reference:
P. Z. Wang et al., ImmuCo: a database of gene co-expression in immune cells. Nucleic Acids Res 43, D1133-D1139 (2015).

## Slide 2
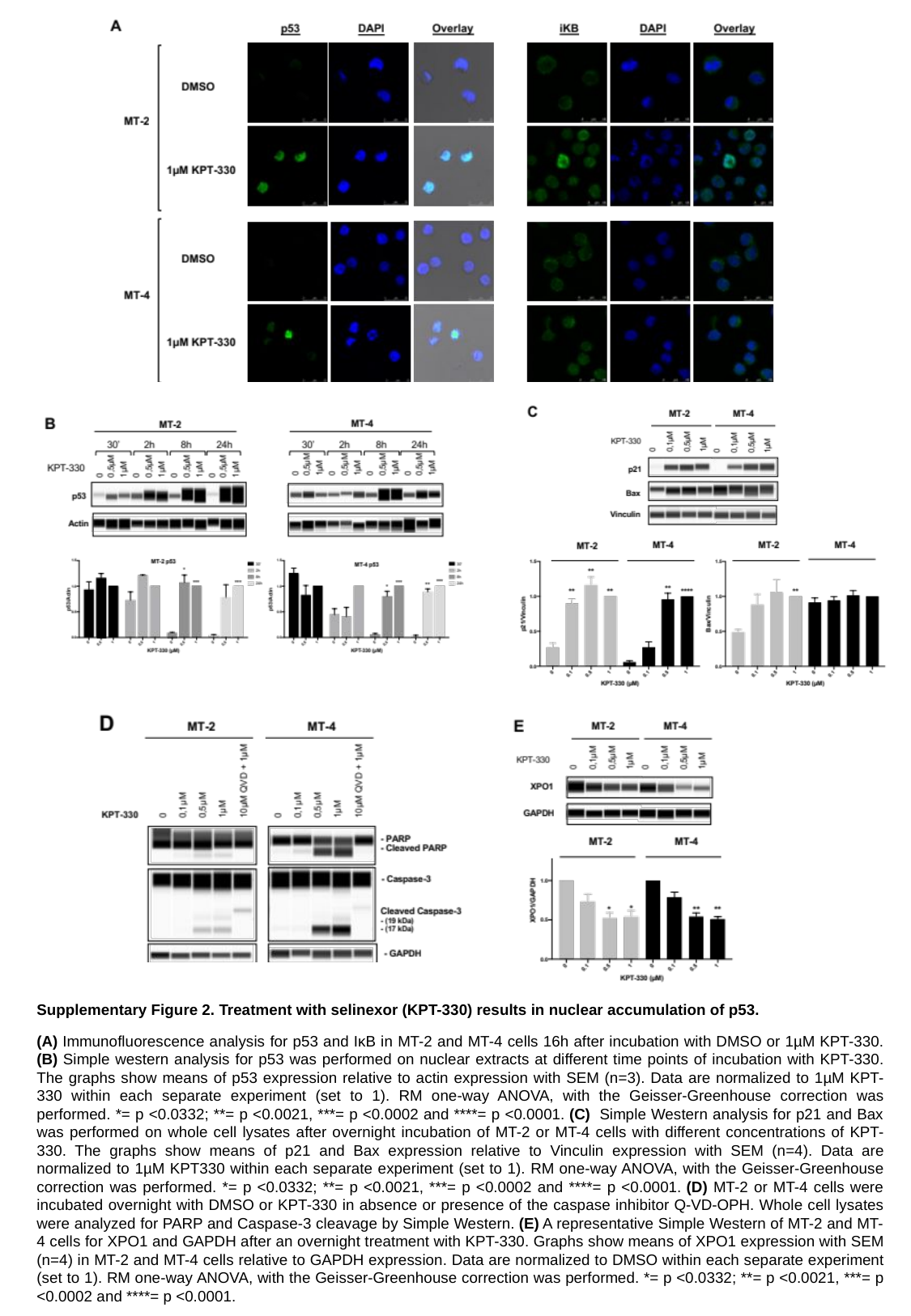

Supplementary Figure 2. Treatment with selinexor (KPT-330) results in nuclear accumulation of p53.
(A) Immunofluorescence analysis for p53 and IκB in MT-2 and MT-4 cells 16h after incubation with DMSO or 1µM KPT-330. (B) Simple western analysis for p53 was performed on nuclear extracts at different time points of incubation with KPT-330. The graphs show means of p53 expression relative to actin expression with SEM (n=3). Data are normalized to 1µM KPT-330 within each separate experiment (set to 1). RM one-way ANOVA, with the Geisser-Greenhouse correction was performed. *= p <0.0332; **= p <0.0021, ***= p <0.0002 and ****= p <0.0001. (C)  Simple Western analysis for p21 and Bax was performed on whole cell lysates after overnight incubation of MT-2 or MT-4 cells with different concentrations of KPT-330. The graphs show means of p21 and Bax expression relative to Vinculin expression with SEM (n=4). Data are normalized to 1µM KPT330 within each separate experiment (set to 1). RM one-way ANOVA, with the Geisser-Greenhouse correction was performed. *= p <0.0332; **= p <0.0021, ***= p <0.0002 and ****= p <0.0001. (D) MT-2 or MT-4 cells were incubated overnight with DMSO or KPT-330 in absence or presence of the caspase inhibitor Q-VD-OPH. Whole cell lysates were analyzed for PARP and Caspase-3 cleavage by Simple Western. (E) A representative Simple Western of MT-2 and MT-4 cells for XPO1 and GAPDH after an overnight treatment with KPT-330. Graphs show means of XPO1 expression with SEM (n=4) in MT-2 and MT-4 cells relative to GAPDH expression. Data are normalized to DMSO within each separate experiment (set to 1). RM one-way ANOVA, with the Geisser-Greenhouse correction was performed. *= p <0.0332; **= p <0.0021, ***= p <0.0002 and ****= p <0.0001.
